# 3-(4-Fluoro­phenyl­sulfin­yl)-2,4,5,6-tetra­methyl-1-benzo­furan

**DOI:** 10.1107/S1600536814014822

**Published:** 2014-06-25

**Authors:** Hong Dae Choi, Pil Ja Seo, Uk Lee

**Affiliations:** a Department of Chemistry, Dongeui University, San 24 Kaya-dong, Busanjin-gu, Busan 614-714, Republic of Korea; b Department of Chemistry, Pukyong National University, 599-1 Daeyeon 3-dong, Nam-gu, Busan 608-737, Republic of Korea

**Keywords:** crystal structure

## Abstract

In the title compound, C_18_H_17_FO_2_S, the dihedral angle between the plane of the benzo­furan ring system (r.m.s. deviation = 0.013 Å) and that of the 4-fluoro­phenyl ring is 74.30 (5)°. In the crystal, weak C—H⋯O and C—H⋯F hydrogen bonds link the mol­ecules into columns extending in direction [100].

## Related literature   

For the crystal structures of related compounds, see: Choi *et al.* (2012 ▶); Seo *et al.* (2011*a* ▶,*b* ▶).
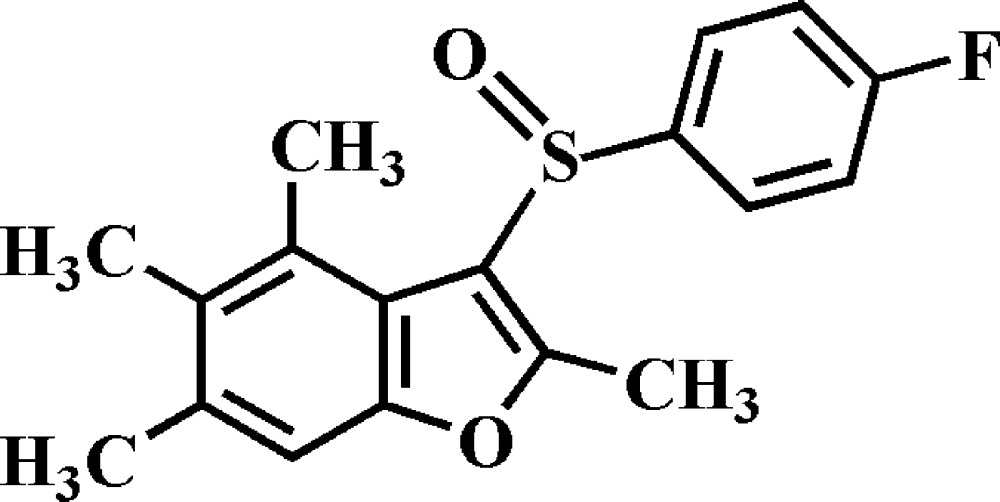


## Experimental   

### 

#### Crystal data  

C_18_H_17_FO_2_S
*M* *_r_* = 316.38
Orthorhombic, 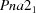

*a* = 7.8869 (2) Å
*b* = 11.0042 (2) Å
*c* = 17.5181 (4) Å
*V* = 1520.38 (6) Å^3^
*Z* = 4
Mo *K*α radiation
μ = 0.23 mm^−1^
*T* = 173 K
0.67 × 0.32 × 0.27 mm

#### Data collection  

Bruker SMART APEXII CCD diffractometer
Absorption correction: multi-scan (*SADABS*; Bruker, 2009 ▶) *T*_min_ = 0.862, *T*_max_ = 0.941
13898 measured reflections
3761 independent reflections
3369 reflections with *I* > 2σ(*I*)
*R*_int_ = 0.039

#### Refinement  

*R*[*F* ^2^ > 2σ(*F* ^2^)] = 0.038
*wR*(*F* ^2^) = 0.094
*S* = 1.05
3761 reflections
203 parameters
1 restraint
H-atom parameters constrained
Δρ_max_ = 0.21 e Å^−3^
Δρ_min_ = −0.25 e Å^−3^
Absolute structure: Flack (1983 ▶), 1812 Friedel pairs
Absolute structure parameter: −0.02 (7)

### 

Data collection: *APEX2* (Bruker, 2009 ▶); cell refinement: *SAINT* (Bruker, 2009 ▶); data reduction: *SAINT*; program(s) used to solve structure: *SHELXS97* (Sheldrick, 2008 ▶); program(s) used to refine structure: *SHELXL97* (Sheldrick, 2008 ▶); molecular graphics: *ORTEP-3 for Windows* (Farrugia, 2012 ▶) and *DIAMOND* (Brandenburg, 1998 ▶); software used to prepare material for publication: *SHELXL97*.

## Supplementary Material

Crystal structure: contains datablock(s) I. DOI: 10.1107/S1600536814014822/cv5468sup1.cif

Structure factors: contains datablock(s) I. DOI: 10.1107/S1600536814014822/cv5468Isup2.hkl

Supporting information file. DOI: 10.1107/S1600536814014822/cv5468Isup3.cml

CCDC reference: 1009827

Additional supporting information:  crystallographic information; 3D view; checkCIF report

## Figures and Tables

**Table 1:** Hydrogen-bond geometry (Å, °)

*D*—H⋯*A*	*D*—H	H⋯*A*	*D*⋯*A*	*D*—H⋯*A*
C15—H15⋯O2^i^	0.95	2.31	3.247 (3)	169
C17—H17⋯F1^ii^	0.95	2.50	3.083 (2)	120

Symmetry codes: (i) 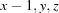; (ii) 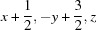.

**Fig. 1.**
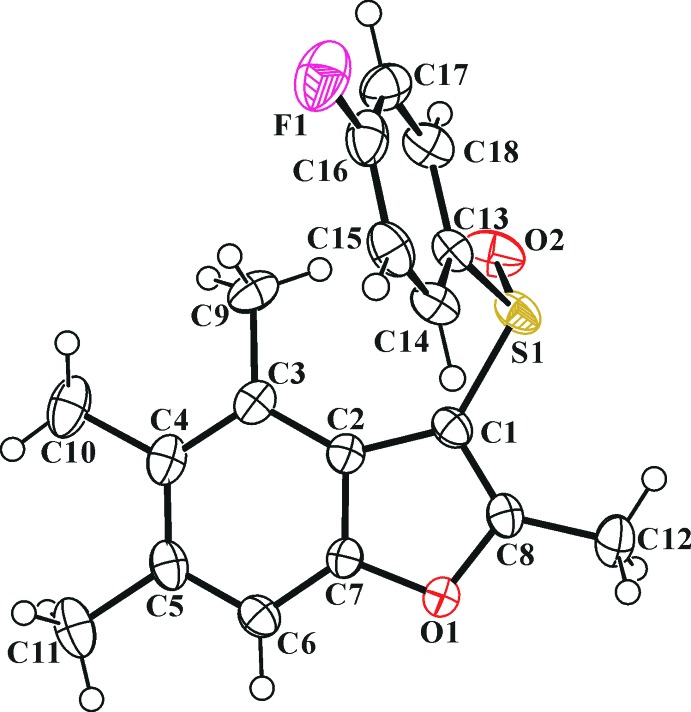
The molecular structure of the title compound with the atom numbering scheme. The displacement ellipsoids are drawn at the 50% probability level. The hydrogen atoms are presented as small spheres of arbitrary radius.

**Fig. 2.**
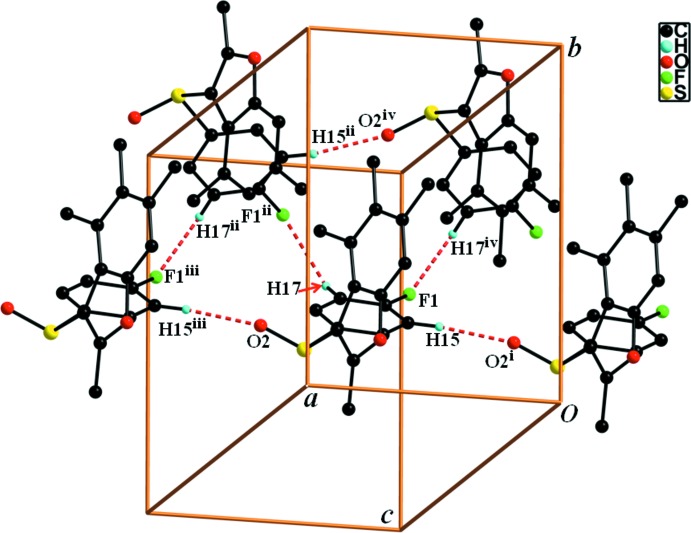
A portion of the crystal packing showing the C—H···O and C—H···F interactions as dotted lines. H atoms non-participating in hydrogen-bonding were omitted for clarity. [Symmetry codes: (i) x - 1, y, z; (ii) x + 1/2, - y + 3/2, z; (iii) x + 1, y, z; (iv) x - 1/2, - y + 3/2, z.]

## supplementary crystallographic information

### S1. Comment

As a part of our ongoing project of 2,4,5,6-tetramethyl-1-benzofuran derivatives containing phenylsulfinyl (Seo *et al.*, 2011*a*), 3-fluorophenylsulfinyl (Seo *et al.*, 2011*b*) and 2-fluorophenylsulfinyl (Choi *et al.*, 2012) substituents in 3-position, we report herein on the crystal structure of the title compound.

In the title molecule (Fig. 1), the benzofuran unit is essentially planar, with a mean deviation of 0.013 (2) Å from the least-squares plane defined by the nine constituent atoms. The 4-fluorophenyl ring is essentially planar, with a mean deviation of 0.002 (1) Å from the least-squares plane defined by the six constituent atoms. The dihedral angle formed by the benzofuran ring system and the 4-fluorophenyl ring is 74.30 (5)°. In the crystal structure (Fig. 2), weak intermolecular C—H···O and C—H···F hydrogen bonds (Table 1) link the molecules into columns extended in [100].

### S2. Experimental

3-Chloroperoxybenzoic acid (77%, 269 mg, 1.2 mmol) was added in small portions to a stirred solution of 3-(4-fluorophenylsulfanyl)-2,4,5,6-tetramethyl-1-benzofuran (330 mg, 1.1 mmol) in dichloromethane (35 mL) at 273 K. After being stirred at room temperature for 5h, the mixture was washed with saturated sodium bicarbonate solution and the organic layer was separated, dried over magnesium sulfate, filtered and concentrated at reduced pressure. The residue was purified by column chromatography (hexane–ethyl acetate, 2:1 *v*/*v*) to afford the title compound as a colorless solid [yield 74%, m.p. 443–444 K; *R*_f_ = 0.59 (hexane–ethyl acetate, 2:1 *v*/*v*)]. Single crystals suitable for X-ray diffraction were prepared by slow evaporation of a solution of the title compound in ethyl acetate at room temperature.

### S3. Refinement

All H atoms were positioned geometrically and refined using a riding model, with C—H = 0.95 Å for aryl and 0.98 Å for methyl H atoms, respectively. *U*_iso_ (H) = 1.2*U*_eq_ (C) for aryl and 1.5*U*_eq_ (C) for methyl H atoms. The positions of methyl hydrogens were optimized using the SHELXL-97's command AFIX 137 (Sheldrick, 2008).

### Crystal data

**Table:**

C_18_H_17_FO_2_S	*D*_x_ = 1.382 Mg m^−^^3^
*M**_r_* = 316.38	Melting point = 444–443 K
Orthorhombic, *P**n**a*2_1_	Mo *K*α radiation, λ = 0.71073 Å
Hall symbol: P 2c -2n	Cell parameters from 3560 reflections
*a* = 7.8869 (2) Å	θ = 2.2–27.1°
*b* = 11.0042 (2) Å	µ = 0.23 mm^−^^1^
*c* = 17.5181 (4) Å	*T* = 173 K
*V* = 1520.38 (6) Å^3^	Block, colourless
*Z* = 4	0.67 × 0.32 × 0.27 mm
*F*(000) = 664	

### Data collection

**Table:**

Bruker SMART APEXII CCD diffractometer	3761 independent reflections
Radiation source: rotating anode	3369 reflections with *I* > 2σ(*I*)
Graphite multilayer monochromator	*R*_int_ = 0.039
Detector resolution: 10.0 pixels mm^-1^	θ_max_ = 28.3°, θ_min_ = 2.2°
φ and ω scans	*h* = −10→10
Absorption correction: multi-scan (*SADABS*; Bruker, 2009)	*k* = −13→14
*T*_min_ = 0.862, *T*_max_ = 0.941	*l* = −23→23
13898 measured reflections	

### Refinement

**Table:**

Refinement on *F*^2^	Secondary atom site location: difference Fourier map
Least-squares matrix: full	Hydrogen site location: difference Fourier map
*R*[*F*^2^ > 2σ(*F*^2^)] = 0.038	H-atom parameters constrained
*wR*(*F*^2^) = 0.094	*w* = 1/[σ^2^(*F*_o_^2^) + (0.0506*P*)^2^ + 0.0839*P*] where *P* = (*F*_o_^2^ + 2*F*_c_^2^)/3
*S* = 1.05	(Δ/σ)_max_ < 0.001
3761 reflections	Δρ_max_ = 0.21 e Å^−^^3^
203 parameters	Δρ_min_ = −0.25 e Å^−^^3^
1 restraint	Absolute structure: Flack (1983), 1812 Friedel pairs
Primary atom site location: structure-invariant direct methods	Absolute structure parameter: −0.02 (7)

### Special details

**Table:**

Geometry. All esds (except the esd in the dihedral angle between two l.s. planes) are estimated using the full covariance matrix. The cell esds are taken into account individually in the estimation of esds in distances, angles and torsion angles; correlations between esds in cell parameters are only used when they are defined by crystal symmetry. An approximate (isotropic) treatment of cell esds is used for estimating esds involving l.s. planes.
Refinement. Refinement of F^2^ against ALL reflections. The weighted R-factor wR and goodness of fit S are based on F^2^, conventional R-factors R are based on F, with F set to zero for negative F^2^. The threshold expression of F^2^ > 2sigma(F^2^) is used only for calculating R-factors(gt) etc. and is not relevant to the choice of reflections for refinement. R-factors based on F^2^ are statistically about twice as large as those based on F, and R- factors based on ALL data will be even larger.

### Fractional atomic coordinates and isotropic or equivalent isotropic displacement parameters (Å^2^)

**Table:**

	*x*	*y*	*z*	*U*_iso_*/*U*_eq_	
S1	0.51230 (6)	0.40717 (5)	0.79515 (3)	0.03465 (13)	
F1	−0.01469 (18)	0.65099 (13)	0.97462 (9)	0.0533 (4)	
O1	0.34363 (18)	0.36849 (11)	0.58751 (7)	0.0285 (3)	
O2	0.67580 (19)	0.47156 (18)	0.80968 (9)	0.0501 (4)	
C1	0.4429 (3)	0.43098 (17)	0.70108 (10)	0.0284 (4)	
C2	0.4022 (2)	0.53931 (16)	0.65691 (10)	0.0258 (4)	
C3	0.4111 (2)	0.66591 (17)	0.66725 (10)	0.0290 (4)	
C4	0.3498 (3)	0.73996 (17)	0.60784 (11)	0.0322 (4)	
C5	0.2879 (3)	0.68941 (18)	0.53945 (11)	0.0320 (4)	
C6	0.2837 (3)	0.56431 (17)	0.52893 (11)	0.0303 (4)	
H6	0.2432	0.5291	0.4829	0.036*	
C7	0.3410 (2)	0.49393 (15)	0.58838 (10)	0.0248 (4)	
C8	0.4051 (2)	0.33292 (16)	0.65686 (10)	0.0292 (4)	
C9	0.4869 (3)	0.7198 (2)	0.73820 (12)	0.0379 (5)	
H9A	0.5548	0.6581	0.7645	0.057*	
H9B	0.3960	0.7478	0.7720	0.057*	
H9C	0.5595	0.7887	0.7244	0.057*	
C10	0.3533 (3)	0.87599 (18)	0.61812 (15)	0.0465 (6)	
H10A	0.4666	0.9068	0.6057	0.070*	
H10B	0.3260	0.8961	0.6712	0.070*	
H10C	0.2696	0.9135	0.5842	0.070*	
C11	0.2277 (3)	0.7687 (2)	0.47404 (14)	0.0458 (6)	
H11A	0.1927	0.7172	0.4312	0.069*	
H11B	0.3202	0.8222	0.4577	0.069*	
H11C	0.1313	0.8181	0.4909	0.069*	
C12	0.4159 (3)	0.19987 (17)	0.66832 (12)	0.0401 (5)	
H12A	0.5087	0.1670	0.6374	0.060*	
H12B	0.3088	0.1620	0.6528	0.060*	
H12C	0.4373	0.1825	0.7223	0.060*	
C13	0.3505 (2)	0.49187 (17)	0.84426 (10)	0.0285 (4)	
C14	0.1820 (3)	0.47592 (19)	0.82583 (10)	0.0318 (4)	
H14	0.1518	0.4272	0.7832	0.038*	
C15	0.0572 (3)	0.53021 (19)	0.86895 (11)	0.0352 (4)	
H15	−0.0593	0.5205	0.8567	0.042*	
C16	0.1075 (3)	0.59918 (18)	0.93053 (12)	0.0360 (5)	
C17	0.2734 (3)	0.61688 (18)	0.94997 (13)	0.0381 (5)	
H17	0.3029	0.6655	0.9928	0.046*	
C18	0.3969 (3)	0.56253 (19)	0.90609 (11)	0.0349 (5)	
H18	0.5132	0.5735	0.9182	0.042*	

### Atomic displacement parameters (Å^2^)

**Table:**

	*U*^11^	*U*^22^	*U*^33^	*U*^12^	*U*^13^	*U*^23^
S1	0.0286 (2)	0.0469 (3)	0.02846 (19)	0.0087 (2)	−0.0041 (2)	0.0047 (2)
F1	0.0522 (9)	0.0416 (8)	0.0662 (9)	0.0090 (6)	0.0207 (7)	−0.0019 (7)
O1	0.0321 (7)	0.0232 (6)	0.0300 (6)	0.0017 (6)	−0.0017 (6)	−0.0008 (5)
O2	0.0249 (7)	0.0827 (12)	0.0429 (9)	0.0025 (8)	−0.0057 (6)	−0.0053 (8)
C1	0.0249 (9)	0.0339 (10)	0.0264 (8)	0.0038 (8)	−0.0003 (7)	0.0030 (7)
C2	0.0251 (10)	0.0258 (8)	0.0265 (8)	−0.0008 (7)	0.0039 (7)	0.0012 (7)
C3	0.0256 (10)	0.0282 (9)	0.0332 (9)	−0.0029 (8)	0.0096 (8)	−0.0016 (7)
C4	0.0300 (11)	0.0260 (9)	0.0406 (10)	−0.0015 (8)	0.0131 (9)	0.0041 (7)
C5	0.0287 (10)	0.0330 (10)	0.0344 (9)	0.0043 (8)	0.0077 (8)	0.0104 (8)
C6	0.0285 (10)	0.0353 (10)	0.0271 (8)	0.0013 (8)	0.0019 (8)	0.0032 (8)
C7	0.0230 (9)	0.0237 (8)	0.0277 (8)	0.0013 (7)	0.0031 (7)	0.0003 (7)
C8	0.0284 (10)	0.0274 (9)	0.0318 (9)	0.0047 (8)	0.0006 (8)	0.0040 (7)
C9	0.0397 (12)	0.0354 (11)	0.0385 (10)	−0.0108 (9)	0.0074 (9)	−0.0103 (9)
C10	0.0478 (14)	0.0286 (10)	0.0631 (14)	−0.0003 (10)	0.0213 (12)	0.0057 (10)
C11	0.0433 (13)	0.0459 (12)	0.0481 (12)	0.0081 (11)	0.0053 (10)	0.0209 (10)
C12	0.0431 (13)	0.0280 (10)	0.0491 (11)	0.0056 (9)	−0.0008 (10)	0.0028 (9)
C13	0.0283 (10)	0.0322 (9)	0.0249 (7)	0.0017 (8)	−0.0009 (8)	0.0055 (7)
C14	0.0288 (10)	0.0411 (11)	0.0255 (8)	−0.0032 (9)	−0.0038 (7)	0.0039 (7)
C15	0.0258 (10)	0.0417 (11)	0.0382 (10)	0.0012 (9)	−0.0017 (8)	0.0119 (9)
C16	0.0404 (13)	0.0286 (9)	0.0390 (10)	0.0021 (9)	0.0114 (9)	0.0078 (8)
C17	0.0432 (13)	0.0345 (10)	0.0366 (9)	−0.0108 (9)	0.0053 (10)	−0.0032 (8)
C18	0.0267 (10)	0.0456 (12)	0.0323 (9)	−0.0081 (9)	−0.0024 (8)	0.0038 (8)

### Geometric parameters (Å, º)

**Table:**

S1—O2	1.4932 (17)	C9—H9C	0.9800
S1—C1	1.7560 (18)	C10—H10A	0.9800
S1—C13	1.799 (2)	C10—H10B	0.9800
F1—C16	1.361 (3)	C10—H10C	0.9800
O1—C8	1.365 (2)	C11—H11A	0.9800
O1—C7	1.381 (2)	C11—H11B	0.9800
C1—C8	1.361 (3)	C11—H11C	0.9800
C1—C2	1.457 (3)	C12—H12A	0.9800
C2—C7	1.387 (2)	C12—H12B	0.9800
C2—C3	1.407 (3)	C12—H12C	0.9800
C3—C4	1.408 (3)	C13—C14	1.379 (3)
C3—C9	1.501 (3)	C13—C18	1.383 (3)
C4—C5	1.408 (3)	C14—C15	1.377 (3)
C4—C10	1.508 (3)	C14—H14	0.9500
C5—C6	1.389 (3)	C15—C16	1.377 (3)
C5—C11	1.517 (3)	C15—H15	0.9500
C6—C7	1.374 (2)	C16—C17	1.366 (3)
C6—H6	0.9500	C17—C18	1.377 (3)
C8—C12	1.480 (3)	C17—H17	0.9500
C9—H9A	0.9800	C18—H18	0.9500
C9—H9B	0.9800		
			
O2—S1—C1	110.99 (10)	C4—C10—H10B	109.5
O2—S1—C13	106.57 (10)	H10A—C10—H10B	109.5
C1—S1—C13	98.65 (9)	C4—C10—H10C	109.5
C8—O1—C7	106.39 (13)	H10A—C10—H10C	109.5
C8—C1—C2	107.33 (16)	H10B—C10—H10C	109.5
C8—C1—S1	118.95 (14)	C5—C11—H11A	109.5
C2—C1—S1	133.56 (14)	C5—C11—H11B	109.5
C7—C2—C3	119.05 (16)	H11A—C11—H11B	109.5
C7—C2—C1	104.00 (15)	C5—C11—H11C	109.5
C3—C2—C1	136.95 (17)	H11A—C11—H11C	109.5
C2—C3—C4	117.44 (17)	H11B—C11—H11C	109.5
C2—C3—C9	121.18 (18)	C8—C12—H12A	109.5
C4—C3—C9	121.36 (17)	C8—C12—H12B	109.5
C3—C4—C5	121.30 (16)	H12A—C12—H12B	109.5
C3—C4—C10	118.68 (19)	C8—C12—H12C	109.5
C5—C4—C10	120.02 (19)	H12A—C12—H12C	109.5
C6—C5—C4	120.84 (17)	H12B—C12—H12C	109.5
C6—C5—C11	117.55 (19)	C14—C13—C18	120.66 (19)
C4—C5—C11	121.59 (18)	C14—C13—S1	120.38 (15)
C7—C6—C5	116.75 (18)	C18—C13—S1	118.57 (15)
C7—C6—H6	121.6	C15—C14—C13	120.33 (18)
C5—C6—H6	121.6	C15—C14—H14	119.8
C6—C7—O1	124.06 (16)	C13—C14—H14	119.8
C6—C7—C2	124.57 (16)	C14—C15—C16	117.6 (2)
O1—C7—C2	111.37 (14)	C14—C15—H15	121.2
C1—C8—O1	110.92 (15)	C16—C15—H15	121.2
C1—C8—C12	133.95 (18)	F1—C16—C17	118.5 (2)
O1—C8—C12	115.13 (16)	F1—C16—C15	118.1 (2)
C3—C9—H9A	109.5	C17—C16—C15	123.4 (2)
C3—C9—H9B	109.5	C16—C17—C18	118.4 (2)
H9A—C9—H9B	109.5	C16—C17—H17	120.8
C3—C9—H9C	109.5	C18—C17—H17	120.8
H9A—C9—H9C	109.5	C17—C18—C13	119.6 (2)
H9B—C9—H9C	109.5	C17—C18—H18	120.2
C4—C10—H10A	109.5	C13—C18—H18	120.2
			
O2—S1—C1—C8	126.77 (17)	C8—O1—C7—C2	0.87 (19)
C13—S1—C1—C8	−121.68 (17)	C3—C2—C7—C6	−1.4 (3)
O2—S1—C1—C2	−58.4 (2)	C1—C2—C7—C6	178.85 (18)
C13—S1—C1—C2	53.1 (2)	C3—C2—C7—O1	178.97 (16)
C8—C1—C2—C7	0.3 (2)	C1—C2—C7—O1	−0.7 (2)
S1—C1—C2—C7	−174.90 (17)	C2—C1—C8—O1	0.2 (2)
C8—C1—C2—C3	−179.3 (2)	S1—C1—C8—O1	176.24 (13)
S1—C1—C2—C3	5.5 (4)	C2—C1—C8—C12	−179.5 (2)
C7—C2—C3—C4	2.7 (3)	S1—C1—C8—C12	−3.5 (3)
C1—C2—C3—C4	−177.7 (2)	C7—O1—C8—C1	−0.6 (2)
C7—C2—C3—C9	−175.95 (18)	C7—O1—C8—C12	179.11 (17)
C1—C2—C3—C9	3.6 (3)	O2—S1—C13—C14	162.46 (14)
C2—C3—C4—C5	−2.4 (3)	C1—S1—C13—C14	47.42 (16)
C9—C3—C4—C5	176.21 (18)	O2—S1—C13—C18	−24.61 (18)
C2—C3—C4—C10	178.38 (18)	C1—S1—C13—C18	−139.66 (16)
C9—C3—C4—C10	−3.0 (3)	C18—C13—C14—C15	−0.1 (3)
C3—C4—C5—C6	0.8 (3)	S1—C13—C14—C15	172.70 (15)
C10—C4—C5—C6	179.98 (19)	C13—C14—C15—C16	−0.5 (3)
C3—C4—C5—C11	−177.67 (19)	C14—C15—C16—F1	−178.51 (17)
C10—C4—C5—C11	1.5 (3)	C14—C15—C16—C17	0.7 (3)
C4—C5—C6—C7	0.6 (3)	F1—C16—C17—C18	178.83 (18)
C11—C5—C6—C7	179.10 (18)	C15—C16—C17—C18	−0.4 (3)
C5—C6—C7—O1	179.29 (17)	C16—C17—C18—C13	−0.2 (3)
C5—C6—C7—C2	−0.2 (3)	C14—C13—C18—C17	0.4 (3)
C8—O1—C7—C6	−178.71 (18)	S1—C13—C18—C17	−172.50 (15)

### Hydrogen-bond geometry (Å, º)

**Table:**

*D*—H···*A*	*D*—H	H···*A*	*D*···*A*	*D*—H···*A*
C15—H15···O2^i^	0.95	2.31	3.247 (3)	169
C17—H17···F1^ii^	0.95	2.50	3.083 (2)	120

Symmetry codes: (i) *x*−1, *y*, *z*; (ii) *x*+1/2, −*y*+3/2, *z*.
